# Burden of early-onset pancreatic cancer and its association with risk factors in women of childbearing age

**DOI:** 10.1371/journal.pone.0356644

**Published:** 2026-08-25

**Authors:** Jiaxing Li, Jing Luo, Qihui Hu, Zhipeng Liu, Yule Luo, Yanbo Li, Paoyong Zou, Rui Tao

**Affiliations:** 1 Department of Hepatobiliary Surgery, Bishan Hospital of Chongqing Medical University, Chongqing, China; 2 Department of Hepatobiliary Surgery, Southwest Hospital of Army Medical University, Chongqing, China; 3 Weight Management Center, Bishan Hospital, Chongqing University of Chinese Medicine, Chongqing, China; SKUMS: Shahrekord University of Medical Science, IRAN, ISLAMIC REPUBLIC OF

## Abstract

**Introduction:**

Early-onset pancreatic cancer (EOPC), diagnosed before age 50, is rising globally. Women of childbearing age (WCBA, 15–49 years) constitute nearly the same population as EOPC patients but have unique metabolic vulnerabilities. The intersection of EOPC trends with WCBA-specific metabolic risks remains underexplored. This study delineates the global EOPC burden among WCBA and its links to core metabolic determinants, providing an evidence base to guide targeted interventions in this priority population and support progress toward international development goals.

**Methods:**

A two-stage design was employed. First, Global Burden of Disease 2023 data were analyzed to assess EOPC mortality and disability-adjusted life years (DALYs) among WCBA (1990–2023), quantifying trends and population attributable fractions (PAFs) for high fasting blood glucose (FBG) and high body mass index (BMI). Second, a retrospective case-control study provided supportive clinical correlation for the identified risk factors.

**Results:**

Globally, absolute EOPC deaths and DALYs in WCBA more than doubled from 1990 to 2023. Age-standardized rates remained stable globally, but increased in low and middle Sociodemographic Index (SDI) regions. High FBG was the largest population-attributable risk factor (global PAF = approximately 64%), with an inverse SDI gradient. High BMI PAF showed an inverted U-shaped distribution. In the supportive clinical analysis, high FBG remained independently associated with EOPC (adjusted OR=4.64; 95% CI: 2.88–7.49), while high BMI lost significance after adjustment, suggesting overlapping metabolic pathways.

**Conclusion:**

Demographic factors (population growth and aging) drive most of the increase in absolute EOPC burden among WCBA. High FBG is the leading modifiable risk factor at the population level, though reverse causation may partly explain its strong association. Targeted glycemic control and metabolic interventions are urgently needed in this population.

## Introduction

Pancreatic cancer is a leading cause of cancer-related mortality globally and currently ranks as the fourth most frequent cause of cancer death. Its incidence and mortality rates have continued to climb in recent years, while the five-year survival rate remains dismal at approximately 10% [1]. The majority of patients are diagnosed at an advanced, incurable stage, and even among those who undergo surgical resection, roughly 80% will eventually succumb to disease recurrence [2,3]. Despite advances in treatment modalities and ongoing clinical investigations, the overall prognosis for pancreatic cancer has seen little improvement.

A particularly concerning trend is the rising incidence of early-onset pancreatic cancer (EOPC), defined as diagnosis before the age of 50, which is now observed in numerous regions worldwide [4]. This increase presents a significant public health challenge [5]. Clinically, EOPC in younger patients is often associated with more aggressive pathological features, such as perineural invasion and poor differentiation, compared to its later-onset counterpart [6,7]. Consequently, a deeper understanding of the disease burden of EOPC and its underlying determinants is of substantial clinical and public health importance.

Metabolic dysregulation, particularly hyperglycemia and obesity, are well-established risk factors for pancreatic cancer. Hyperglycemia can promote tumor cell proliferation and inhibit apoptosis by activating signaling cascades such as the RAS-MAPK pathway via insulin and insulin-like growth factor-1 (IGF-1) [8]. Separately, obesity contributes to a pro-carcinogenic microenvironment; the secretion of pro-inflammatory cytokines like IL-6 and TNF-α from visceral adipose tissue activates pancreatic stellate cells, fostering fibrosis and chronic inflammation [9]. These two factors often act synergistically, exacerbating insulin resistance and inflammation, thereby amplifying the risk of pancreatic carcinogenesis [10,11].

The United Nations Sustainable Development Goals (SDG3) aim to reduce maternal mortality and improve non-communicable disease prevention. In this context, the health of women of childbearing age (WCBA, defined as 15–49 years) is paramount—not only for maternal outcomes but also for their own cancer risk [12]. WCBA are particularly vulnerable to metabolic disturbances due to physiological changes in the reproductive years. Conditions such as gestational diabetes mellitus and polycystic ovary syndrome increase the prevalence of hyperglycemia and insulin resistance in this age group, potentially elevating their susceptibility to EOPC. Because EOPC (diagnosed before age 50) and WCBA (aged 15–49) are essentially the same population, focusing on WCBA allows us to highlight reproductive-age metabolic risks that may be overlooked in general EOPC studies [13,14]. Epidemiological evidence supports this, indicating that WCBA with glycemic abnormalities or obesity face a significantly increased risk of pancreatic cancer [15]. Therefore, in the context of the global epidemics of diabetes and obesity, implementing targeted metabolic health management and EOPC risk prevention strategies for WCBA is of critical practical importance.

Despite this overlap, systematic investigations into the burden of EOPC specifically framed around WCBA metabolic vulnerabilities remain scarce, particularly in resource-limited settings. This knowledge gap prevents adequate attention from being directed toward this high-risk group and may hinder progress toward relevant public health objectives. With the global burden of metabolic diseases continuing to escalate [16], a systematic evaluation of the epidemiological characteristics of EOPC in WCBA and its association with high fasting blood glucose (FBG) and high body mass index (BMI) is urgently needed to inform the development of stratified prevention and intervention strategies.

To address this research gap, the present study adopted a two-stage design. In the first stage, we utilized data from the Global Burden of Disease (GBD) 2023 study to systematically assess the global burden of EOPC among WCBA from 1990 to 2023, stratifying by Sociodemographic Index (SDI). We also quantified the population-attributable risk of key metabolic factors. In the second stage, we conducted a retrospective case-control study to provide supportive clinical correlation for the primary metabolic risk factors identified in the GBD analysis, acknowledging that the clinical data are hypothesis-generating rather than confirmatory. The findings are intended to provide a robust scientific foundation for early screening and targeted interventions for EOPC in this vulnerable population.

## Methods

### Analysis of global burden of disease data

#### Data sources and definitions.

Data were obtained from the GBD 2023 study, coordinated by the Institute for Health Metrics and Evaluation. This study integrates global data sources including vital registries, surveys, hospital records, and literature, applying standardized modeling to produce comparable disease burden estimates. The study population comprised women aged 15–49 years globally. EOPC was defined as pancreatic cancer diagnosed before age 50. Pancreatic cancer was defined using ICD-10 code C25. Key metrics included deaths and disability-adjusted life years (DALYs). Age-standardized rates (ASRs) per 100,000 population with 95% uncertainty intervals (UIs) were calculated to facilitate cross-population comparisons [17].

#### Stratification by sociodemographic index.

Countries were stratified using the Sociodemographic Index (SDI), a composite measure of per-capita income, mean education, and fertility rate. Regions were categorized as high, high-middle, middle, low-middle, and low SDI [18].

#### Trend and decomposition analysis.

Temporal trends were assessed using estimated annual percentage change (EAPC) derived from a log-linear regression model: ln(ASR) = α + β(year), where EAPC = 100 × (e^β − 1) [19]. Joinpoint regression was applied to identify significant inflection points in the trend. The maximum number of joinpoints was set to 5. Model selection was performed using a permutation test with a significance level of α = 0.05. For each segment, the annual percentage change was calculated, and the average annual percentage change for the entire period 1990–2023 was also computed [20–22].

A demographic‑epidemiological decomposition method was used to partition the absolute change in EOPC burden into three components: population growth, population aging, and epidemiological changes. The decomposition formula is as follows: the total change in the number of deaths from time t_1_ to t_2_ equals the sum of the contribution of population growth, the contribution of changes in age structure, and the contribution of changes in age‑specific mortality rates. Using a stepwise replacement approach, each component was estimated independently, and the sum of the three components equals the net observed change in death counts [23].

#### Predictive modeling and risk attribution.

A Bayesian Age-Period-Cohort (BAPC) model with integrated nested Laplace approximation (INLA) and second-order random walk priors for age and period projected EOPC burden through 2050. We performed out-of-sample validation by training the model on 1990–2015 data and comparing predictions to observed 2016–2023 data. The mean absolute percentage error was approximately 12%, indicating acceptable calibration [24].

Population attributable fractions for metabolic risk factors were obtained directly from the GBD Comparative Risk Assessment framework. Specifically, the PAF for each risk factor was extracted from the GBD 2023 results database and not independently recalculated. The GBD framework computes PAF using the formula: PAF = P × (RR − 1) / (1 + P × (RR − 1)), where P is the exposure prevalence in the population and RR is the relative risk for the risk‑outcome pair derived from meta‑analyses, incorporating a theoretical minimum risk exposure level. Because the GBD database does not publicly release the disaggregated values of P and RR for each specific cause and population subgroup, we provide the exact query parameters used for extraction to ensure reproducibility.

### Clinical validation study

#### Study design and participants.

A retrospective case-control design provided supportive clinical correlation for key metabolic risk factors. Cases were female pancreatic cancer patients aged 15–49 years diagnosed January 2010–October 2025, with histopathological confirmation. The data for this study were accessed on 3 December 2025. Controls were healthy women aged 15–49 years undergoing routine physical examinations during the same period. Exclusion criteria included other malignancies, severe organ dysfunction, or >10% missing key variables. After exclusions, 122 cases and 366 controls were included, with a 1:3 frequency matching by age (±2 years only; no matching on socioeconomic status or comorbidities). Sample size was estimated: assuming a two-sided α = 0.05, power = 0.8, a case-to-control ratio of 1:3, a control exposure prevalence of 25% for high FBG, and an expected odds ratio of 2.0, the minimum required sample size was approximately 100 cases and 300 controls. To allow for a margin and improve statistical power, we ultimately included 122 cases and 366 controls.

#### Variable definitions and data collection.

Demographic data, height, weight, and FBG were collected. BMI was calculated as weight/height^2^ (kg/m^2^). FBG was measured using glucose oxidase. Based on WHO Asian population recommendations, high BMI was defined as ≥25 kg/m^2^ and hyperglycemia as FBG ≥ 6.1 mmol/L [25]. Data on smoking, alcohol consumption, pancreatitis history, and family history of pancreatic cancer were also collected. Quality control included standardized data extraction manuals, researcher training, dual independent data entry, and random verification of 20% of records. Missing data: Cases with >10% missing values on key variables were excluded. For remaining missing values (<5% per variable), listwise deletion was applied.

### Statistical analysis

Analyses were performed using SPSS 26.0. Categorical data were compared using χ² tests. Univariate and multivariate logistic regression assessed associations with EOPC. To avoid bias from automated variable selection, multivariable models included a pre-specified set of confounders based on literature: age, high FBG, high BMI, smoking history (binary), pancreatitis history, and family history of pancreatic cancer. No stepwise selection was used. Multicollinearity was assessed using variance inflation factors (VIFs); all VIFs were <2.5, indicating no significant collinearity. The Hosmer-Lemeshow test was used for goodness-of-fit. Adjusted odds ratios (ORs) and 95% confidence intervals (CIs) were calculated. Statistical significance was set at P < 0.05.

This study was reviewed and approved by the Ethics Committee of Bishan Hospital of Chongqing Medical University (Approval No. cqbyky11-20251130-01). The requirement for informed consent was waived due to the retrospective nature of the study.

## Results

The findings are presented in two sections. The first section, derived from GBD data, delineates the global burden, temporal trends, and metabolic risk attribution of EOPC among WCBA from 1990 to 2023. The second section validates the association of key risk factors with EOPC through a clinical case-control study.

### Global disease burden and trends of EOPC among WCBA

#### Descriptive analysis of disease burden.

From 1990 to 2023, the absolute numbers of deaths and DALYs due to EOPC among WCBA increased significantly worldwide. The number of deaths rose from 104,169 in 1990–256,770 in 2023, and DALYs increased from 2,342,183–5,249,084. However, the age-standardized mortality rate (ASMR) and DALY rate remained relatively stable during this period. The ASMR increased slightly from 5.03 to 5.15 per 100,000, while the age-standardized DALY rate decreased marginally from 109.13 to 107.59 per 100,000 (Table 1).

**Table 1:** The number, ASR, and EAPC for deaths and DALYs due to EOPC among WCBA in 1990 and 2023 globally.

Location	Measure	1990		2023		EAPC (95% CI)1990–2023
		Number (95% UIs)	ASR (95% UIs)	Number (95% UIs)	ASR (95% UIs)	
Global	Deaths	104169.12	5.03	256769.7	5.15	0.07*
		(94617.18 - 112161.23)	(4.54 - 5.42)	(223921.74 - 280182.81)	(4.5 - 5.62)	(0.04 to 0.10)
High SDI		84512.75	6.71	188757.61	7.23	0.27*
		(77005.6 - 89885.1)	(6.11 - 7.14)	(162534.67 - 205455.52)	(6.32 - 7.81)	(0.23 to 0.30)
High-middle SDI		12047.83	3.47	34520.65	3.21	−0.23*
		(10453.97 - 13816.22)	(3.01 - 3.99)	(30218.77 - 39177.75)	(2.81 - 3.65)	(−0.31 to −0.16)
Middle SDI		3167.35	1.9	14341.73	3.13	1.35*
		(2528.38 - 4030.31)	(1.51 - 2.41)	(11540.01 - 17715.03)	(2.53 - 3.86)	(1.28 to 1.41)
Low-middle SDI		2238.28	1.24	9727.5	2.03	1.28*
		(1597.15 - 3052.52)	(0.88 - 1.68)	(7419.88 - 12729.31)	(1.56 - 2.66)	(1.19 to 1.37)
Low SDI		2056.82	1.18	9137.47	1.98	1.11*
		(1356.43 - 2992.56)	(0.78 - 1.71)	(6375.33 - 12428.63)	(1.39 - 2.7)	(0.96 to 1.25)
China		17569.38	4.09	44881.65	3.69	−0.27*
		(14285.7 - 21859.09)	(3.33 - 5.11)	(36239.55 - 57511.86)	(2.99 - 4.69)	(−0.44 to −0.10)
Global	DALYs	2342182.99	109.13	5249083.62	107.59	−0.16*
		(2139391.77 - 2529011.41)	(99.67 - 117.74)	(4726126.94 - 5679425.24)	(96.81 - 116.47)	(−0.19 to −0.12)
High SDI		1812349.37	147.84	3577783.19	150.16	−0.03
		(1667844.25 - 1928945.99)	(136.37 - 157.49)	(3205187.87 - 3859676.61)	(136.09 - 161.4)	(−0.07 to 0.00)
High-middle SDI		320380.96	84.29	787160.89	73.03	−0.66*
		(278360.84 - 366425.09)	(73.07 - 96.47)	(698565.67 - 888737.61)	(64.73 - 82.3)	(−0.74 to −0.57)
Middle SDI		86712.34	46.82	372423.43	76.87	1.49*
		(69426.5 - 110540.62)	(37.43 - 59.55)	(297882.31 - 461254.61)	(61.56 - 95.21)	(1.43 to 1.56)
Low-middle SDI		60672.15	29.61	249508.44	48.86	1.51*
		(43065.57 - 82810.25)	(21.1 - 40.48)	(189314.92 - 326890.81)	(37.12 - 63.99)	(1.41 to 1.60)
Low SDI		58819.07	29.41	256349.65	49.48	1.33*
		(38654.08 - 85559.22)	(19.32 - 42.82)	(179125.64 - 347267.81)	(34.55 - 67.06)	(1.08 to 1.58)
China		478087.56	103.99	984617.41	82.47	−1.16*
		(393468.85 - 584507.56)	(85.37 - 127.85)	(824039.31 - 1222324.53)	(69.13 - 101.35)	(−1.31 to −1.00)

Note: *P < 0.05.

Burden distribution varied markedly by SDI. In 2023, high SDI regions accounted for the largest share of absolute burden (73.5% of deaths, 68.1% of DALYs). However, age-standardized rates exhibited upward trajectories in middle, low-middle, and low SDI regions during the study period. In China (a high-middle SDI country), ASMR fell from 4.09 to 3.69 per 100,000 and DALY rate from 103.99 to 82.47 per 100,000, outpacing global average declines.

Geographic disparities in 2023 were pronounced. Elevated burden clustered in Eastern Europe and Central Asia, whereas parts of South, Southeast, and East Asia showed lower rates (S1 Table).

#### Trend analysis.

Globally, ASMR for EOPC demonstrated a slight but significant increase from 1990 to 2023 (EAPC = 0.07; 95% CI: 0.04–0.10), while DALY rate showed a modest decline (EAPC = −0.16; 95% CI: −0.19 to −0.12) (Table 1).

Regional trends diverged. High SDI regions experienced rising mortality (EAPC = 0.27) but stable DALY rates (EAPC = −0.03). High-middle SDI regions registered substantial declines in both metrics (mortality EAPC = −0.23; DALY EAPC = −0.66). Alarmingly, middle, low-middle, and low SDI regions all exhibited significant upward trends, signaling a rapidly escalating threat. China continued its favorable trajectory with declines in both mortality (EAPC = −0.27) and DALY rates (EAPC = −1.16).

#### Joinpoint regression analysis.

**Fig 1:**
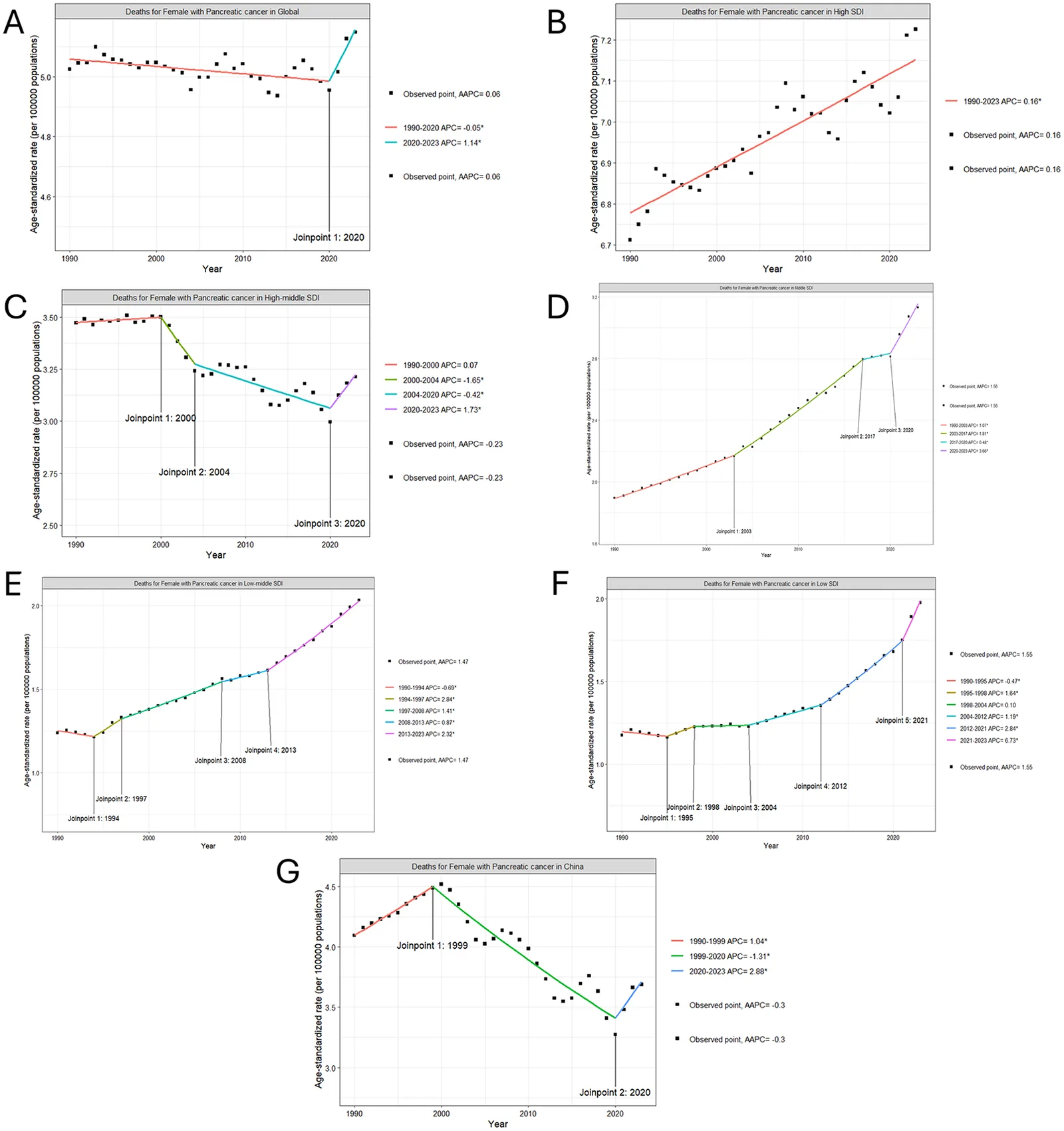
Joinpoint regression analysis on the ASR of deaths due to EOPC among WCBA. (A) global, (B) high SDI, (C) high-middle SDI, (D) middle SDI, (E) low-middle SDI, (F) low SDI, (G) China.

**Fig 2:**
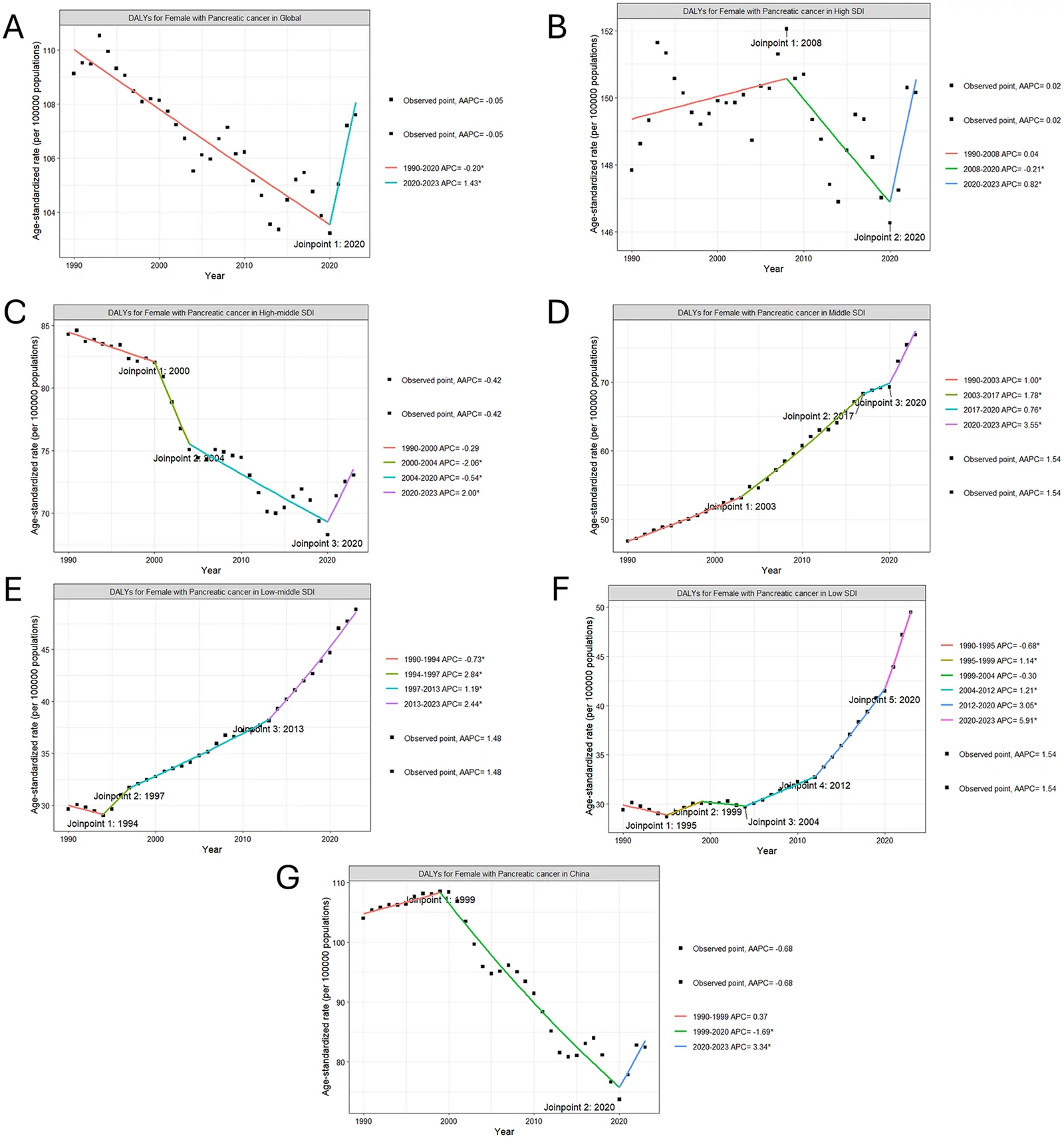
Joinpoint regression analysis on the ASR of DALYs due to EOPC among WCBA. (A) global, (B) high SDI, (C) high-middle SDI, (D) middle SDI, (E) low-middle SDI, (F) low SDI, (G) China.

Global ASMR showed a slight upward trend (AAPC = 0.06), while DALY rate declined minimally (AAPC = −0.05). China demonstrated consistent decreases (ASMR AAPC = −0.30; DALY AAPC = −0.68) (Figs 1, 2**)**.

Regional patterns were heterogeneous. In high SDI regions, ASMR increased steadily (AAPC = 0.16). High-middle SDI regions saw declines (ASMR AAPC = −0.23; DALY AAPC = −0.42). Middle, low-middle, and low SDI regions exhibited significant increases, with middle SDI showing the fastest growth (mortality AAPC = 1.56). Notably, low SDI experienced a sharp acceleration after 2012, with mortality APC reaching 6.73 during 2021–2023, becoming the region with the most rapid burden escalation.

#### Decomposition analysis.

**Fig 3:**
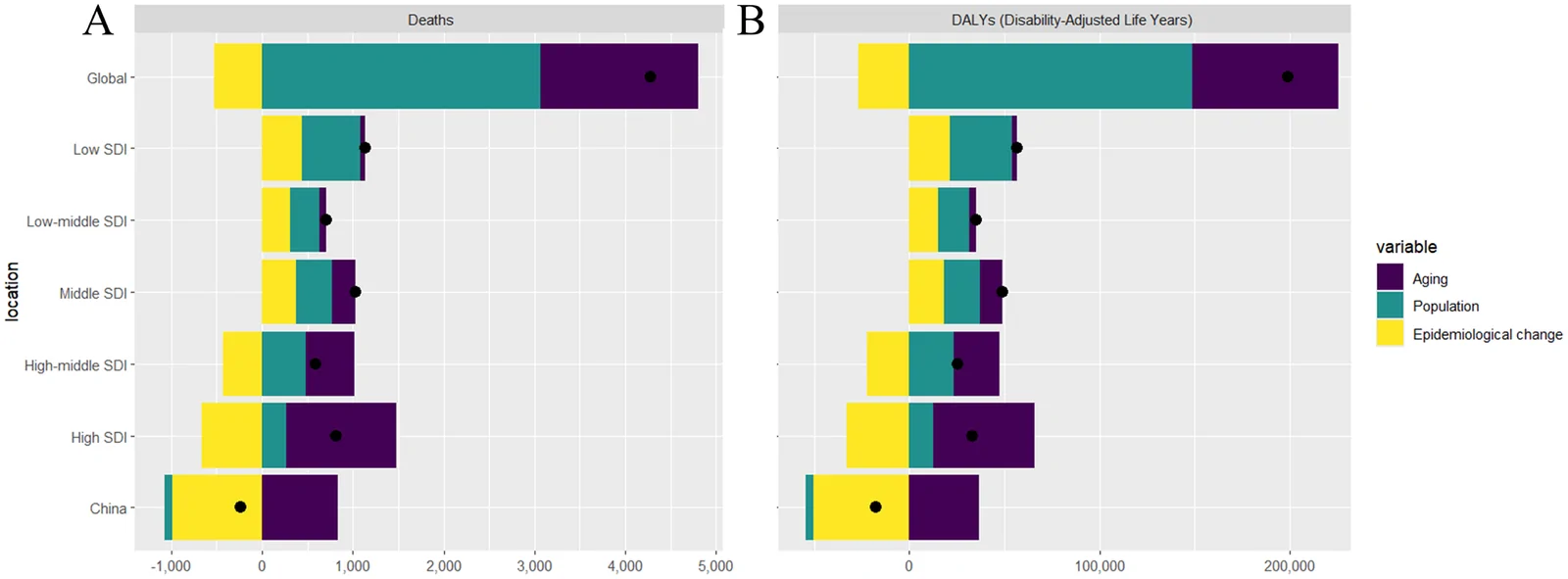
Decomposition analysis on the ASR of deaths and DALYs due to EOPC among WCBA in global, 5 SDI regions, and China. (A) deaths, (B) DALYs.

Globally, the rise in absolute EOPC deaths and DALYs from 1990 to 2023 was driven primarily by population growth (contributing 71.77% and 74.80%, respectively) and secondarily by aging (40.60% and 38.66%) (Fig 3). In China, epidemiological shifts emerged as the predominant factor, driving a substantial reduction in both deaths (421.05%) and DALYs (284.02%) that effectively counteracted the increase attributable to population aging. The observed percentages exceeding 100% reflect the fact that favorable epidemiological trends—such as improved glycemic control—more than offset the net positive change, thereby yielding a modest overall decline; consequently, these component contributions are quantified relative to this small net change. Across SDI strata, aging predominated in high and high-middle regions, while middle, low-middle, and low SDI faced dual pressures from population growth and adverse epidemiological shifts.

#### Predictive analysis to 2050.

**Fig 4:**
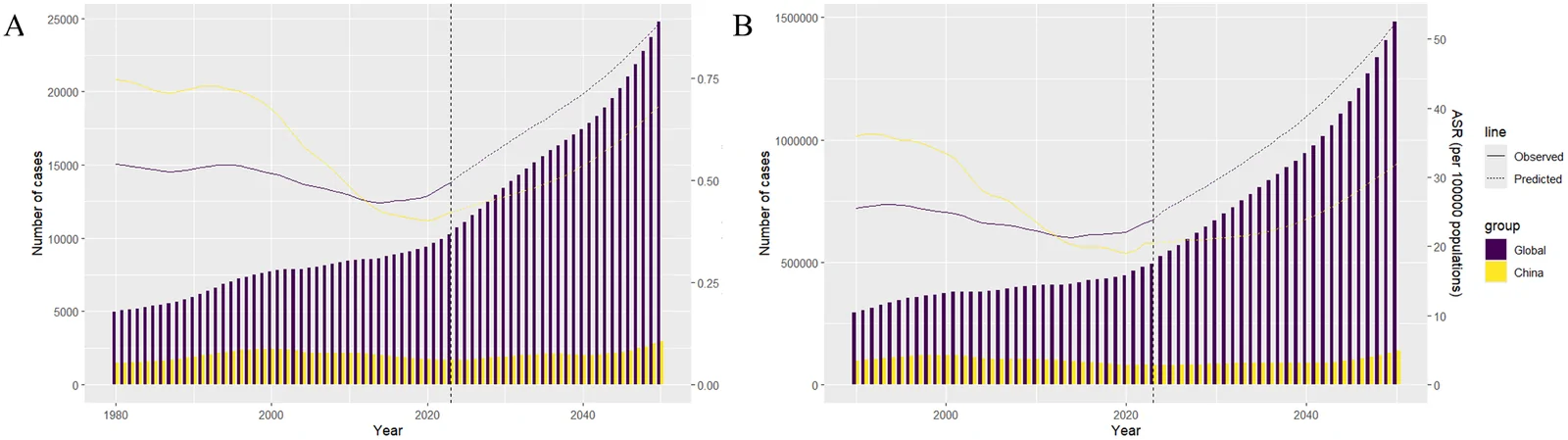
Predictive analysis on the deaths and DALYs of EOPC among WCBA to 2050. (A) deaths, (B) DALYs.

BAPC model projections indicated continued global increases in both absolute numbers and age-standardized rates of EOPC deaths and DALYs through 2050, with the projections shown alongside 80% and 95% credible intervals (Fig 4). China also showed projected rises in age-standardized mortality and DALY rates.

#### Attributable fraction of metabolic risk factors.

**Fig 5:**
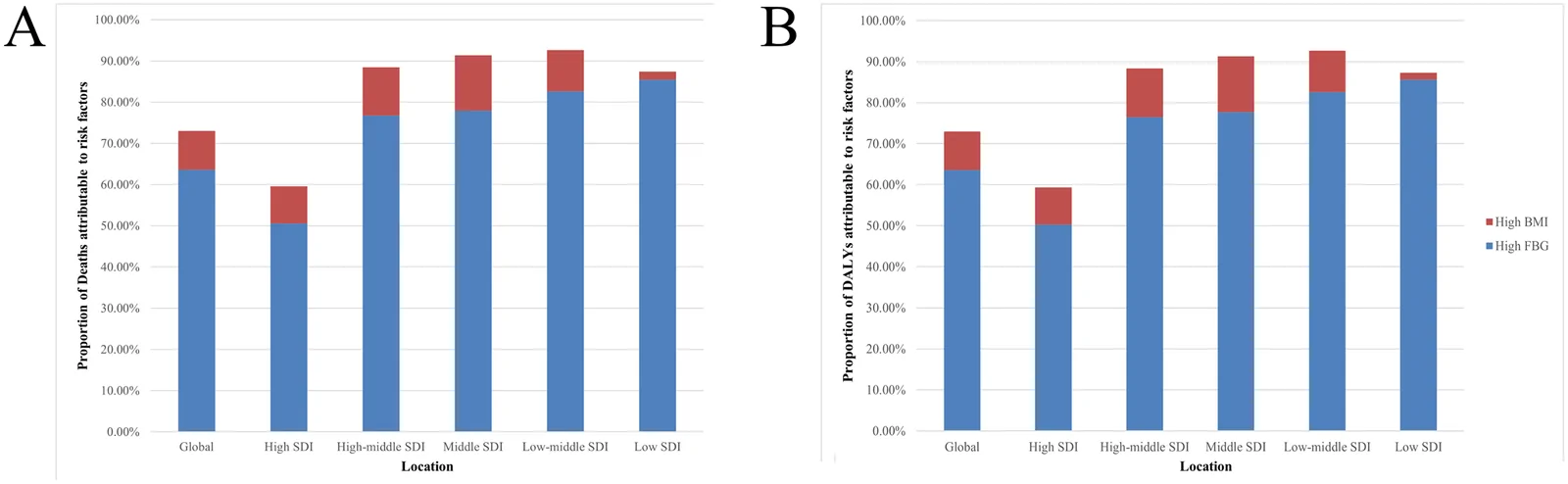
The proportion of deaths and DALYs attributable to each risk factor for EOPC among WCBA in 2023. (A) deaths, (B) DALYs. Note: PAFs are independent estimates for each risk factor and do not sum to 100%.

In 2023, high FBG was the largest population-attributable metabolic risk factor for EOPC among WCBA globally, with PAFs of approximately 64% for deaths and DALYs. High BMI contributed about 9% for both deaths and DALYs (Fig 5). These PAFs should be interpreted cautiously due to potential reverse causation (pancreatic cancer–induced hyperglycemia) and GBD modeling assumptions.

PAFs exhibited a marked SDI gradient. High FBG-attributable burden was inversely related to SDI, peaking in low SDI regions (deaths: about 85%; DALYs: about 86%) and lowest in high SDI (deaths: about 51%). In contrast, high BMI-attributable PAF followed an inverted U-shaped distribution, highest in middle SDI (deaths: about 13%) and lowest in low SDI (deaths: about 2%).

### Clinical validation study results

#### Baseline characteristics.

The clinical validation included 488 women (122 cases, 366 controls) with comparable age distributions (median 46 years, IQR 42–47). No significant differences emerged between groups for alcohol consumption or pregnancy history (P > 0.05). However, cases exhibited significantly higher proportions of high BMI, high FBG, smoking history, family history of pancreatic cancer, and history of pancreatitis (all P < 0.05) (Table 2).

**Table 2:** Baseline characteristics and logistic regression analysis of risk factors for EOPC among WCBA.

Variable	Control group (N = 366) n (%)	Case group (N = 122) n (%)	Univariate analysis OR (95% CI)	P value	Multivariate analysis Adjusted OR (95% CI)	P value
High BMI	144 (39.3)	66 (54.1)	1.817 (1.202–2.747)	0.005	1.170 (0.729–1.880)	0.515
High FBG	76 (20.8)	68 (55.7)	4.805 (3.102–7.442)	<0.001	4.642 (2.878–7.489)	<0.001
Smoking history	9 (2.5)	8 (6.6)	2.784 (1.050–7.383)	0.04	3.1 (1.049–9.162)	0.041
Alcohol consumption history	26 (7.1)	12 (9.8)	–	0.331	–	–
Family history of pancreatic cancer	6 (1.6)	8 (6.6)	4.211 (1.431–12.389)	0.009	5.219 (1.640–16.606)	0.005
History of pancreatitis	4 (1.1)	9 (7.4)	7.208 (2.178–23.850)	0.001	7.723 (2.125–28.073)	0.002
Pregnancy history	318 (86.9)	108 (88.5)	–	0.638	–	–

#### Risk factor analysis for EOPC.

Univariate logistic regression revealed strong associations with EOPC for high FBG (OR = 4.805), high BMI (OR = 1.817), smoking (OR = 2.784), family history (OR = 4.211), and pancreatitis history (OR = 7.208) (all P < 0.05). Alcohol and pregnancy history were not significant.

In multivariate analysis adjusting for all prespecified confounders (age, high FBG, high BMI, smoking, pancreatitis history, family history), high FBG remained a potent independent risk factor (adjusted OR = 4.642). History of pancreatitis (adjusted OR = 7.723) and family history of pancreatic cancer (adjusted OR = 5.219) also retained independent significance. Smoking history remained significant (adjusted OR = 3.100). Notably, high BMI lost statistical significance after adjustment (adjusted OR = 1.170; 95% CI: 0.729–1.880; P = 0.515).

The attenuation of the BMI effect after adjustment for FBG suggests that these two metabolic factors share overlapping pathways; however, formal mediation analysis was not performed, and causality cannot be inferred. Sensitivity analysis using a stricter hyperglycemia definition (FBG ≥ 7.0 mmol/L) yielded consistent results, with high FBG remaining significantly associated with EOPC (aOR = 4.76, 95% CI: 2.60–8.71, P < 0.001). Furthermore, after false discovery rate (FDR) correction for multiple testing, high FBG remained statistically significant (q < 0.001). Variance inflation factors (VIFs) for all variables were <2.5, indicating no substantial multicollinearity. The Hosmer-Lemeshow test indicated acceptable model fit (P = 0.4037).

## Discussion

This two-stage study systematically evaluated the global burden of EOPC among WCBA, emphasizing the contributions of high FBG and high BMI as key metabolic risk factors. GBD analysis revealed that while absolute EOPC burden rose substantially worldwide between 1990 and 2023, age-standardized rates remained relatively stable. High FBG emerged as the largest population-attributable metabolic factor, though reverse causation likely contributes to this association. A supportive clinical analysis identified high FBG as independently associated with EOPC, whereas high BMI’s association attenuated after multivariable adjustment. These findings offer critical insights for developing targeted prevention strategies in this vulnerable population.

The observed stability in global age-standardized EOPC mortality among WCBA, contrasted with rising absolute numbers, indicates that demographic forces, particularly population growth and aging, are the primary drivers of the expanding burden. This “population-driven” pattern aligns with recent analyses of global cancer trends [26]. However, substantial heterogeneity across SDI levels warrants particular attention. Middle and low SDI regions confront dual pressures from population expansion and escalating epidemiological risk, consistent with the broader shift of cancer burden toward developing nations [27]. This disparity likely reflects rapid lifestyle transitions in these regions, characterized by diets rich in sugar and fat alongside declining physical activity, which collectively increase exposure to metabolic risk factors. Concurrently, limited healthcare infrastructure may delay diagnosis and restrict treatment access, further exacerbating outcomes. By quantifying these drivers through decomposition analysis, our study provides granular evidence for understanding EOPC burden dynamics across developmental contexts.

This investigation confirms the pivotal role of high FBG in EOPC pathogenesis at both population and individual levels. GBD data demonstrated that high FBG accounted for the highest PAF among metabolic factors for global EOPC deaths in WCBA, reaching approximately 64% overall and exceeding 85% in low SDI regions. Our clinical case-control study further substantiates this association (adjusted OR = 4.642). The peak attributable risk in low SDI regions suggests that uncontrolled hyperglycemia may represent a substantial yet underrecognized burden in areas with weak diabetes screening and management infrastructure.

The relationship between high BMI and EOPC proved more complex. At the population level, high BMI contributed 9.4% to global EOPC burden, peaking in middle SDI regions with an inverted U-shaped distribution. This pattern may reflect transitional phases in the global obesity epidemic: rapid obesity escalation in middle SDI regions contrasts with high SDI areas where, despite elevated prevalence, medical intervention and public health awareness may partially mitigate impact; low SDI regions have not yet entered peak obesity phases [28]. However, in clinical multivariate analysis, the independent effect of high BMI disappeared after FBG adjustment. This aligns with prospective studies where obesity-pancreatic cancer associations weakened following diabetes adjustment [29], suggesting that BMI-related EOPC risk may operate primarily through inducing metabolic disturbances—insulin resistance, hyperinsulinemia, and chronic inflammation—with high FBG serving as a core biochemical marker of these processes. Consequently, clinical and public health strategies should prioritize systematic glycemic monitoring and comprehensive metabolic management for obese individuals rather than focusing solely on weight reduction.

This study also reaffirmed pancreatitis history, family history of pancreatic cancer, and smoking as independent EOPC risk factors. The strong pancreatitis-EOPC association (adjusted OR = 7.723) aligns with classic “inflammation-cancer” sequence theory. Recent research further implicates dysregulated immune cells and cytokine networks within the pancreatitis microenvironment in promoting carcinogenesis [30,31]. Identification of these non-metabolic risk factors underscores the need for diversified prevention strategies. WCBA with pancreatitis history or family predisposition should be considered high-risk and receive enhanced surveillance even when metabolic parameters appear normal.

### Strengths and limitations

The principal strength of this study lies in its two-stage design, enabling cross-validation between macro-epidemiological and micro-clinical data, thereby enhancing conclusion robustness. Nevertheless, several limitations merit consideration. First, GBD estimates rely on modeling assumptions and broad uncertainty intervals, particularly in data‑sparse regions, and the potential for ecological fallacy limits individual‑level inferences. Second, tumor‑induced hyperglycemia likely creates reverse causation that inflates the population attributable fractions for high fasting blood glucose in both the GBD and clinical datasets. Third, the clinical study has several limitations. The modest sample size (n = 122) and matching only on age limit statistical power and introduce potential healthy volunteer bias. Key variables were coarsely defined: smoking as binary (no pack‑years), the pancreatitis odds ratio was unstable due to few cases, and pregnancy history was recorded only as yes/no, lacking information on parity, age at first pregnancy, or gestational diabetes status—despite the study’s focus on women of childbearing age. The use of Asian‑specific cutoffs restricts global generalizability. Additionally, the long study period (2010–2025) saw rising diabetes prevalence and evolving diagnostic practices in China; this temporal heterogeneity was not adjusted for and may affect the findings. Consequently, the non‑significant BMI result should not be interpreted as a true null effect. Fourth, residual confounding from factors such as diet, physical activity, and socioeconomic status cannot be excluded. Finally, projections extending to 2050 assume the continuation of recent trends and do not incorporate the potential impact of future medical breakthroughs or policy shifts. Future research should establish large-scale prospective cohorts to dynamically assess cumulative risk factor effects during early life and reproductive years.

## Conclusion

This two-stage study demonstrates that the absolute global burden of EOPC among WCBA has doubled over three decades, though age-standardized rates remain stable—except in low and middle SDI regions where they are rising. Demographic factors (population growth and aging) drive most of the increase in absolute EOPC burden among WCBA. High FBG is the leading modifiable risk factor at the population level, accounting for approximately 64% of deaths globally and exceeding 85% in low-resource settings, though reverse causation may partly explain this strong association. Clinical supportive analysis confirmed an independent association (OR=4.64) while the association of high BMI attenuated after adjustment, suggesting overlapping metabolic pathways. These findings underscore the urgent need for targeted glycemic control strategies in WCBA, particularly in lower-income regions, alongside enhanced surveillance for those with pancreatitis or family history. Prospective studies are warranted to refine risk stratification and guide early intervention.

## Supporting information

S1 Table The ASR for deaths and DALYs due to EOPC among WCBA in 2023 globally. (XLSX)

S2 Data Supplementary de-identified dataset for the clinical validation study. (SAV)
